# Validity of the Food Frequency Questionnaire Assessing the Folate Intake in Women of Reproductive Age Living in a Country without Food Fortification: Application of the Method of Triads

**DOI:** 10.3390/nu9020128

**Published:** 2017-02-13

**Authors:** Milica Zekovic, Marija Djekic-Ivankovic, Marina Nikolic, Mirjana Gurinovic, Dusanka Krajnovic, Marija Glibetic

**Affiliations:** 1 Centre of Research Excellence in Nutrition and Metabolism, Institute for Medical Research (IMR), University of Belgrade, Tadeusa Koscuska 1, 11158 Belgrade, Serbia; djekic.ivankovic@gmail.com (M.D.-I.); marina.nikolic12@yahoo.com (M.N.); mirjana.gurinovic@gmail.com (M.G.); mglibetic@gmail.com (M.G.); 2 Department of Social Pharmacy and Pharmaceutical Legislation, Faculty of Pharmacy, University of Belgrade, Vojvode Stepe 450, 11000 Belgrade, Serbia; dusica.krajnovic@pharmacy.bg.ac.rs

**Keywords:** folate, FFQ, validation, women, method of triads

## Abstract

The study aimed to examine the external validity of the Folate Food Frequency Questionnaire (F-FFQ) designed for assessing the folate intake in Serbian women of reproductive age. The F-FFQ was tested against repeated 24 h dietary recalls and correspondent nutritional biomarkers (red blood cells (RBC) and serum folate concentrations) using the method of triads. In a cross sectional study, 503 women aged 18–49 years completed dietary questionnaires and representative validation subsample (*n* = 50) provided fasting blood samples for biomarker analyses. Correlation coefficients were calculated between each of the dietary methods and three pair-wise correlations were applied for the calculation of validity coefficients. Correlation coefficients observed between F-FFQ and three 24 h recalls were *r* = 0.56 (*p* < 0.001) and *r* = 0.57 (*p* < 0.001) for total sample and validation group, respectively. Bland–Altman plot and cross-classification analyses indicated good agreement between methods. High validity coefficients were determined between the true intake (I) and dietary assessment methods, F-FFQ (Q) and 24 h dietary recalls (R) (ρQI_rbc_ = 0.871 and ρQI_ser_ = 0.814; ρRI_rbc_ = 0.652 and ρRI_ser_ = 0.698), and moderate ones for biomarkers (B) (ρBI_rbc_ = 0.428 and ρBI_ser_ = 0.421). The F-FFQ is valid instrument for the assessment of dietary folate intake in women living in Serbia, a country without mandatory folic acid food fortification.

## 1. Introduction

Nutritional imbalances during pregnancy can influence gene expression and cause abnormalities of fetal phenotype. Scientific progress in the comprehension of congenital anomalies has led to the conclusion that optimally balanced maternal diet with adequate intake of macro- and micronutrients can contribute to reducing the incidence of these disorders [1]. For the prevention of adverse pregnancy outcomes and normal fetal development folate, water-soluble B_9_ vitamin, is recognized as a nutrient of particular importance [2].

The term folate refers to a group of related compounds including folates naturally present in foods and the synthetic, fully oxidized form, folic acid. The biological functions of folate as a co-enzyme are essentially based on single-carbon units transfer in the processes of purine and pyrimidine nucleotides biosynthesis and metabolism of amino acids methionine, serine, glycine and histidine [3]. Since the pteridine cycle, an element of the folate structure, cannot be synthesized de novo in mammalian body, adequate amount of these compounds with a critical role in numerous biochemical processes must be provided through food and/or supplementation [4]. Unlike naturally occurring folate vitamers, which are labile and prone to losing biological activity during storage, food processing and preparation, folic acid retains stability and is therefore successfully used in the form of supplements and for food fortification [5,6].

There is substantial scientific evidence that maintaining adequate folate status before conception and during the first trimester of pregnancy significantly reduces the risk of occurrence and recurrence of neural tube defects (NTD) [2,7]. Given that the closure of the neural tube, fundamental for the proper formation of the nervous system, occurs during the third and fourth week (i.e., from Day 21 to Day 28) from conception, and that up to 41% of pregnancies are unplanned, it is clear that throughout this period the majority of women are still not, or have only become aware of the pregnancy [8,9]. For this reason, it is crucial to ensure optimal folate intake and status in all women of childbearing age.

Recommendation for optimal dietary intake of naturally-occurring mixed forms of folate for adults is 400 µg according to US Institute of Medicine (IOM) and World Health Organization/Food and Agricultural Organization of the United Nations (WHO/FAO) Expert Consultation group [10]. These organizations emphasize that, in addition to healthy diet, due to problematic stability and bioavailability of food folate, for optimal NTD prevention, it is necessary to provide 400 µg of folic acid daily by supplementation when planning pregnancy or throughout childbearing age [11]. Since it is difficult to attain general compliance with advice on nutrition and supplement usage, many countries worldwide have introduced controlled and strictly regulated folic acid food fortification. In Serbia, such policy has not been implemented and availability of voluntarily fortified food is limited. Furthermore, prior studies indicate that the intake of folate in this country is bellow values recommended by authorities [12], while only 3.9% of women report taking folic acid supplements during periconceptional period [13]. Concern regarding folate inadequacy and associated health issues is widespread across Europe. Majority of European countries lack fortification policy and current strategies, based primarily on dietary counseling and promotion of folic acid supplementation, have not had appreciable public health effect on reducing the prevalence of suboptimal folate intake and NTDs [14,15,16,17].

In facing the challenge of achieving folate adequacy, the first step is reliable and objective nutritional assessment performed with standardized and validated instruments. To address this concern, we developed the Food Frequency Questionnaire for folate intake assessment (F-FFQ). Food Frequency Questionnaires are used to assess usual diet during a defined period of time in a simple and cost-effective manner with relatively small burden imposed on researchers and respondents. Due to aforementioned advantages, this instrument is commonly used in nutritional epidemiological studies all over the world. However, it is important to highlight that a universal FFQ, which could be applied for all population groups and all research questions, does not exist. Demographic, socio-economic, geographical, climatic, cultural and medical status factors all have influence on the diet and FFQ must be created or adapted in accordance with the characteristics of a particular study population [18]. Furthermore, in order to ensure proper interpretation of data obtained by FFQ, it is important to determine the association between reported intakes from the FFQ and true dietary intake [19].

The aim of the present study is to determine whether the F-FFQ is valid tool for assessing the dietary intake of folate in Serbian women of reproductive age.

## 2. Materials and Methods

In this study, the relative ability of the F-FFQ to estimate folate intake was tested against reference method (repeated 24 h dietary recalls), and correspondent biomarkers of folate intake (concentration of folate in serum and red blood cells (RBC)) using the method of triads [20,21]. This triangular approach relies on the availability of quantitative intake information from three methods and uses the correlations between each of them to calculate the validity coefficient. The validity coefficient represents the correlation between the dietary intake reported by the F-FFQ and the unknown true dietary intake. Given that FFQ and 24 h recalls commonly share errors related to misreporting, the main benefit of applying the triads method is the inclusion of biomarkers—objective and independent indicators of nutrient intake [20,21,22]. This approach enables broadening of validation parameters and more comprehensive analyses.

### 2.1. Study Participants

In a one-year period, from June 2014 to July 2015, a cross-sectional study was conducted as a part of a national integrated project with an objective to estimate dietary intake and biomarkers of folate status among women of reproductive age in Serbia. During the recruitment process, flyers with the invitation for participation in the study were available in selected primary health care facilities (community pharmacies and health centers) and educational institutions throughout country. Recruitment material contained detailed information regarding the purpose and objectives of the research, study protocol, as well as rights and expectations of the potential participants. The inclusion criteria for the study were: female sex, age between 18 and 49 years and a regular menstrual cycle. Exclusion criteria comprised: pregnancy, breast feeding, use of drugs that interact with folate metabolism, hormonal substitution and menopause.

**Figure 1:**
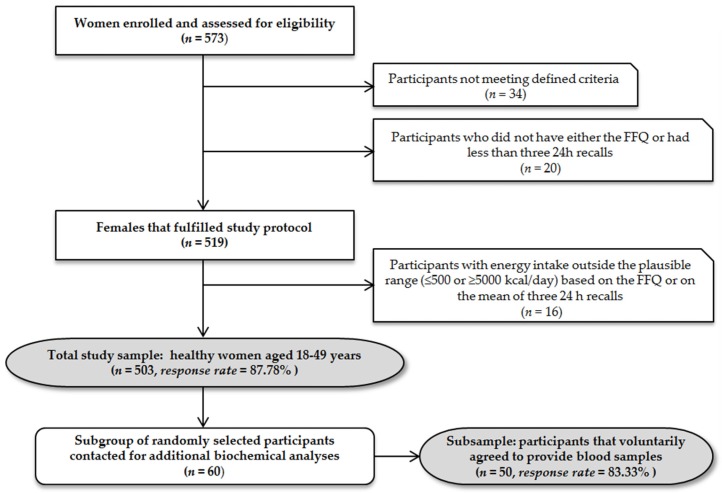
Recruitment process and sample overview (FFQ, Food Frequency Questionnaire).

Recruitment process and sample overview are presented in Figure 1.

### 2.2. Anthropometry

Anthropometry included height and weight measurement of participants dressed only in light clothing. Height was measured to the nearest 0.1 cm (Perspective Enterprises, Kalamazoo, MI, USA) and weight to the nearest 0.1 kg (TBF-300, Tanita Corp., Tokyo, Japan). Body Mass Index (BMI) was calculated as weight (kg)/height-squared (m^2^) [23].

### 2.3. Food Frequency Questionnaire

The FFQ for folate (F-FFQ) was developed by combining a validated FFQ for folate in Croatia region [24] with the NCI/Block Health Habits and History Questionnaire [25]. Further adaptation of the F-FFQ included folate-rich traditional foods consumed in Serbia (such as baked beans “prebranac”, stuffed dock leaves, ajvar/pindjur (traditional roasted red pepper spread), sun-dried peppers stuffed with beans, and dock/spinach pie) (Table S1). Face and content validity of the questionnaire were assessed by an expert panel consisting of five researchers from the Center of Research Excellence in Nutrition and Metabolism in Serbia. The questionnaire was pilot tested for clarity and format improvement among 20 women of reproductive age who did not participate in the main study.

The F-FFQ was designed to capture habitual intake over the previous three months and was self-administered in the presence of trained dietitians. Photographs of food portion sizes (small, medium, and large) were included in the FFQ. In addition to pre-specified portion size options, there was a possibility for respondents to determine usual portion in an open-ended manner. The questionnaire included the following frequency of consumption options: “never”, “once per month”, “2–3 times per month”, “once a week”, “2–3 times per week”, “4–6 times per week” and “every day”. In the case respondents consumed certain food more than once per day they were instructed to specify portion that corresponds to daily intake. All the reported frequencies for ninety food items listed in F-FFQ were converted to frequencies per day with reference to a base value of 1.0 for the “every day” option. Estimates of amounts of food consumed per day were calculated by multiplying the daily equivalent frequency of consumption of food items by the chosen portion size.

In addition, to enhance interpretation of F-FFQ estimates, general questions related to age, education, previous medical conditions, detailed vitamin supplements and medication use and lifestyle habits regarding smoking, physical activity/exercise, alcohol as well as coffee and tea consumption were also incorporated in questionnaire.

### 2.4. Twenty-Four-Hour Dietary Recalls

Three 24 h dietary recalls per participant were performed by multiple pass during the last two weeks of the period covered by the F-FFQ. Interviews were conducted on nonconsecutive days, with two of the recalls being on weekdays and one on a weekend day. Survey calendar was defined so that adequate proportion of weekdays is captured on a group level. Within the structured interview participants reported the complete consumption of food and beverages in preceding 24 h. Data regarding the type of the food or dishes, time and place of consumption, cooking or processing method and the amounts consumed were recorded in suitable survey form in chronological order. All the interviews were done face to face and led by a trained professional according to standardized protocol. The estimated time of data collection was 15–30 min. In order to improve the accuracy of the portion estimate, the questionnaires were administered in conjunction with Food Atlas [26,27]. This amount estimation tool contains color photographs of various portions of foods and dishes whose selection was made on the basis of previously conducted national studies. For each item four to nine serving sizes, measured using calibrated digital scale, were available. All photographs were made in a standardized, uniform manner, with identical lighting, background and shooting distance, so that comparative factors (e.g., plate, cup and cutlery of defined dimensions) were clearly shown as an aid for selection.

### 2.5. Dietary Data Assessment

Quantitative food consumption data, obtained with both instruments (i.e., 24 h dietary recall and FFQ), were processed with DIET ASSESS & PLAN (DELTA Electronic Ltd., Subotica, Serbia), advanced dietary intake assessment and nutrition planning software tool, which has been applied previously in national, regional and international nutritional surveys and evaluated in European Food Safety Authority (EFSA) trial ring [26,27,28,29]. Nutrient intake calculation was performed using the Serbian Food Composition Database, harmonized with EuroFIR standards and embedded in EuroFIR Food Platform and Balkan food platform [30]. For dietary supplement users, content specified by manufacturer and reported information regarding dosage, consumption frequency and duration were taken into account. Total folate intake was estimated in µg dietary folate equivalents (DFE)/day using formula: µg of DFE = [µg of food folate + (1.7 × µg of synthetic folic acid)] and related to recommended values proposed by WHO—Estimated Average Requirements (EAR; 320 µg/day) and Recommended Nutrient Intake (RNI; 400 µg/day) for adults [10].

### 2.6. Biochemical Assessment

Within two days after completing the F-FFQ, blood samples of fifty randomly selected participants were collected by trained medical staff via venipuncture. Prior to specimen collection participants fasted overnight and analyses were performed immediately. Samples were collected in plastic Serum Separator Tube and in tripotassiumethylendiaminetetraacetic acid (K_3_ EDTA) tube (BD Vacutainer; Becton, Dickenson and Company, Plymouth, United Kingdom) for serum and RBC folate concentration analyses, respectively. Biomarkers were determined using the ARCHITECT Folate kit (Abbott Laboratories, Abbott Park, IL, USA), based on Chemiluminescent Microparticle Immunoassay (CMIA) technology and traceable to the Folate WHO International Standard 03/178, on Architect i2000 analyzer (Abbott Laboratories, Abbott Park, IL, USA). Special attention was paid to keeping samples, calibrators and controls protected from light. Prior to RBC hemolysate preparation hematocrit of the EDTA specimen was determined according to the manufacturer’s instructions [31]. The accuracy of the assay was verified with three level control materials (ARCHITECT Folate Controls, Abbott Laboratories, Abbott Park, IL, USA) and all were within manufacturer’s specified range. All assays were done in duplicate. The intra-assay coefficients of variation for folate serum and RBC were 4.3% and 4.7%, while the inter-assay coefficients of variation were 6.1% and 6.3%, respectively.

Based on cut-off values proposed by WHO, folate serum concentrations <6.8 nmol/L were related to folate deficiency, concentrations between 6.8 nmol/L and 13.4 nmol/L to possible deficiency, and values >13.5 nmol/L to adequate status [32]. RBC folate concentrations below 340 nmol/L were considered indicative for folate deficiency and concentrations ≥906 nmol/L as optimal for achieving the greatest reduction of risk for NTD-affected pregnancy [33].

### 2.7. Statistical Analyses

The normality of the data distribution was analyzed using Shapiro–Wilk test for the total sample and the validation group separately. When data were not normally distributed, values were log transformed before analyses. For all parameters, differences between the total sample and validation group were tested using the Wilcoxon Rank Sum test.

Several statistical techniques were applied to evaluate validity of the F-FFQ. Pearson correlation coefficients were calculated to assess association between dietary folate intake estimated by F-FFQ and 24 h recalls on crude and energy-adjusted data for both total sample and validation subgroup [34]. Agreement between methods was further examined by classification of the variables into the quartiles (i.e., in four groups divided by 25th percentile, median and 75th percentile). We calculated the quartiles of folate intake assessed by F-FFQ for the validation group and cross-tabulated these with respective quartiles of 24 h-recall-derived estimates and biomarker levels. Rank of the variable that corresponds to the Pth percentile in the sample was calculated using formula (P/100)(1 + *n*), where *n* is the number of observations. Since the number of participants in validation subsample in our study was 50 calculated ranks were not integer. Therefore, they were rounded to the nearest rank [35]. Discordance and agreement in quartile ranking was assessed as the percentage classification in the same, same or adjacent, opposite (first and third or second and fourth quartile) and absolutely opposite quartile (grossly misclassified—first and fourth quartile). Linear regression analyses were performed to test for significant linear trends between folate intake assessed by F-FFQ, 24 h recalls and folate biomarkers, adjusting for research settings.

Furthermore, to visualize agreement between folate intake results obtained from F-FFQ and 24 h recalls the Bland–Altman plot was constructed [36]. The arithmetic difference in folate intakes between the two methods for each individual was plotted against the average estimation of the two methods. The 95% limit of agreement was calculated as the mean difference ±1.96 standard deviation (SD).

The comprehensive triangular approach to validation, known as the method of triads, was applied as well. Validity coefficient (VC) for the FFQ was calculated using the correlations between the FFQ and the biomarker (r_QB_), 24 h recalls and the biomarker (r_BR_) and the FFQ and 24 h recalls (r_QR_) as shown in the following equation:

ρ_QI =_ √(r_QB_ × r_QR_/r_BR_)

This method has been described in detail by Ocke and Kaaks [20]. Correlation between the biomarker and the FFQ and calculated validity coefficient were used as lower and upper limits of validity coefficients. The 95% confidence intervals for the validity coefficients were estimated using bootstrap sampling where 1000 samples of equal size (*n* = 50) were obtained with replacement from the study subjects [20,37].

A *p* value < 0.05 was considered statistically significant. All statistical analyses were performed using R software package (R Foundation for Statistical Computing, Vienna, Austria) [38].

### 2.8. Ethical Approval

This study was conducted in accordance with the guidelines laid down in the Declaration of Helsinki and all procedures involving human subjects were approved by the Institute for Medical Research Ethics Committee in Serbia (EO112/2015). Written informed consent for inclusion was obtained from all participants.

## 3. Results

The average age of participants was 34.09 (SD = 10.74) years. Distributed in age groups, 88, 179, 159 and 78 of the studied women were 18–25, 25–35, 35–45 and 45–49 years old, respectively. Total sample and validation group were not statistically different in weight (65.09 (SD = 8.15) kg vs. 62.24 (SD = 6.06) kg), height (168.89 (SD = 5.21) cm vs. 169.94 (SD = 4.42) cm), BMI (22.83 (SD = 2.64) kg/m^2^ vs. 21.99 (SD = 1.93) kg/m^2^) and waist to hip ratio (0.77 (SD = 0.06) vs. 0.76 (SD = 0.04)).

According to anthropometric measurements, 4.17% of the women were classified in the category of underweight (BMI < 18.5 kg/m^2^), 20.08% in overweight (BMI = 25.0–29.9 kg/m^2^) and 5.57% in obese (BMI ≥ 30.0 kg/m^2^) while 70.18% had BMI within normal range (BMI = 18.5–24.9 kg/m^2^) [23]. At the time of the survey, 2.58% of the participants had primary education, 58.65% secondary and 38.77% post-secondary education. Consumption of folic acid in the form of single or multivitamin/multivitamin–multimineral supplements at least once a week was reported by 4.77% of women. Smoking habit was reported by 29.22% of participants in total sample and 26.04% in validation group. Based on F-FFQ self-reports, among 503 studied women 41.75% consumed alcohol on a weekly basis, 89.66% consumed coffee and 63.61% tea at least a cup a day. Moderate physical activity between 30 min and 3.5 h weekly was stated by 76.34% of women in the main sample and 77.73% in validation subgroup.

Estimated daily energy, macronutrient and folate intake together with correlations between the estimates of the dietary intakes by the F-FFQ and 24 h recalls are presented in Table 1. Pearson correlation coefficients between folate intake assessed by F-FFQ and 24 h dietary recalls did not substantially change after adjustment for total energy intake based on residual model, neither for total sample (crude data: *r* = 0.56, *p*-value < 0.001 vs. energy-adjusted data: *r* = 0.53, *p*-value < 0.001) nor for validation subgroup (crude data: *r* = 0.57, *p*-value < 0.001 vs. energy-adjusted data *r* = 0.59, *p*-value < 0.001). There were no significant differences in intake estimates between the total sample and validation group (*p* > 0.05). Only 6.16% and 6.76% of all the participants reached folate EAR benchmark while 96.22% and 93.64% had folate intakes bellow recommended 400 µg DFE/day based on repeated 24 h recalls and FFQ, respectively.

In Table 2 the median values and 5th and 95th percentiles of the daily intake levels of twelve food groups and the corresponding relative contributions of these food groups to the daily folate intake based on 24 h dietary recalls are presented. Major folate food sources, identified for total 503 women using both dietary methods, are presented in Table 3.

Mean folate serum level in the studied women was 12.29 (SD = 6.59) nmol/L and RBC folate 438.66 (SD = 144.63) nmol/L. Using a cut-off value of 6.8 nmol/L for serum folate and 340 nmol/L for RBC folate, the prevalence of blood folate levels indicative for folate deficiency was 30% (*n* = 15) and 24% (*n* = 12), respectively. Adequate folate serum concentrations were observed in 38% (*n* = 19) of studied participants. None of the women met or exceeded 906 nmol/L, the value of RBC folate associated with lowest risk of having NTD-affected pregnancy. A significant increase of serum and RBC folate concentrations were identified with an increase of folate intake (*p* < 0.05; Table 4).

**Figure 2:**
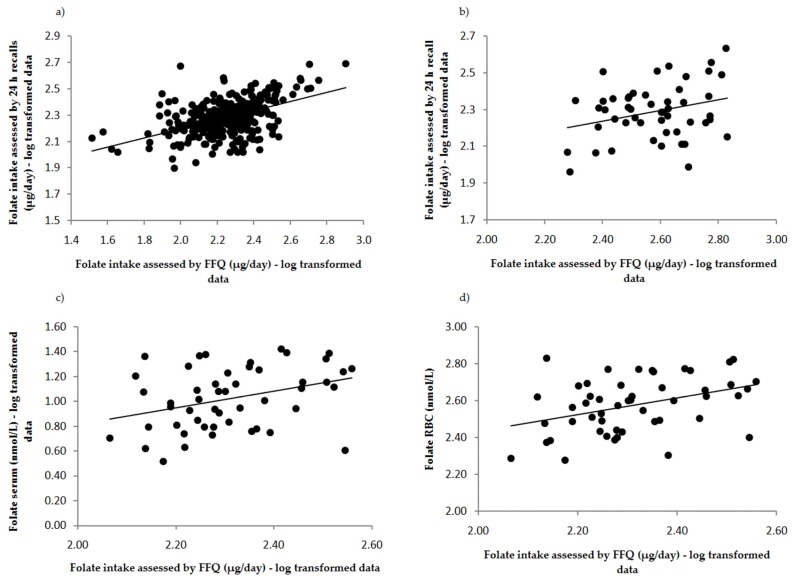
Correlations between folate intake assessed by the F-FFQ and the average of 24 h recalls in: (**a**) total sample (*n* = 503); and (**b**) validation subsample (*n* = 50); and folate intake assessed by the FFQ and: (**c**) folate serum concentration; and (**d**) red blood cells (RBC) folate concentration among Serbian women of reproductive age. All correlations were significant: (**a**) *r* = 0.56, *p* < 0.001; (**b**) *r* = 0.57, *p* < 0.001; (**c**) r_ser_ = 0.28, *p* < 0.01; and (**d**) r_rbc_ = 0.37, *p* < 0.01.

**Figure 3:**
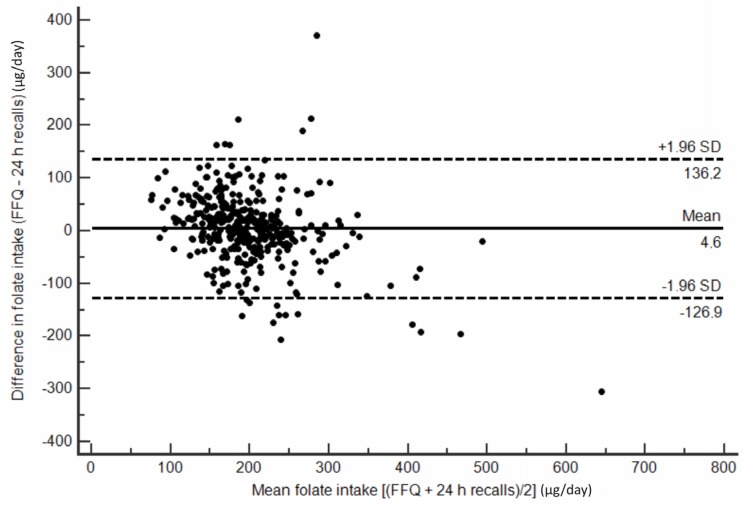
Bland–Altman plot assessing the agreement between the F-FFQ and the average of three 24 h dietary recalls for estimating folate intake, Serbian women of reproductive age. For each participan, the difference in folate intakes between the two methods was plotted against the mean folate intake by two methods: solid line, mean difference; and dotted line, 95% limits of agreement (LOA).

The ability of F-FFQ to classify participants into the correct quartile of folate intake assessed by 24 h recalls and biochemical indices is summarized in Table 5. The F-FFQ classified more than 80% of subjects correctly or closely as 24 h recalls and folate biomarkers. Figure 2 illustrates correlations between the F-FFQ, 24 h dietary recalls and biomarkers of folate status (i.e., serum and RBC folate). Furthermore, Bland–Altman plots indicated good agreement between methods, as less than 5% (precisely 20 cases, i.e., 3.98%) of cases fell beyond the limits of agreement (Figure 3).

**Figure 4:**
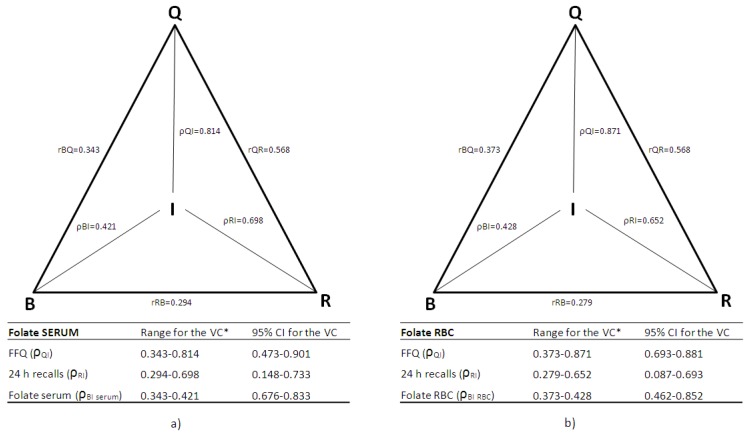
Triangular relationship of the correlation coefficients (r_QB_, r_RB_, r_QR_) between folate intake estimated by the FFQ (Q), the average of the three 24 h dietary recalls (R) and the biomarkers (B) of folate status: (**a**) folate concentration in serum; and (**b**) red blood cells (RBC) folate concentration, and validity coefficients (VC: ρ_QI_, ρ_BI_, ρ_RI)_ between true intake (I) and estimated intakes, with 95% confidence intervals. * The lower limit of the true validity coefficient is correlation between the biomarker and the two dietary methods (F-FFQ and 24 h recalls), and the upper limit is calculated by the method of triads.

Application of the method of triads enabled triangular comparisons of the correlation coefficients between F-FFQ, reference method (repeated 24 h recalls) and biochemical measurements—serum folate (Figure 4a) and RBC folate level (Figure 4b). The validity coefficient of the F-FFQ was high for both serum and RBC folate, indicating a strong relationship between true folate dietary intake and F-FFQ estimates (Figure 4a,b).

## 4. Discussion

Nutritional status assessment is nowadays recognized as an important part of medical care at individual and population level. In order to generate reliable information in the sphere of nutrition research, the use of valid, comprehensive instruments adapted to specific characteristics of the population of interest is essential. The aim of the present study was to validate the Food Frequency Questionnaire for the assessment of folate intake (F-FFQ) among women of reproductive age in Serbia, a country without mandatory folic acid food fortification. Therefore, we collected dietary intake data by applying two methods (24 h dietary recalls and FFQ), determined concentrations of two biochemical indicators of folate status and applied the method of triads [20]. Validity coefficients for F-FFQ were high regardless of biomarker used (ρQI_rbc_ = 0.871 and ρQI_ser_ = 0.814). Moreover, a high level of agreement between the F-FFQ and 24 h dietary recalls was determined with Bland–Altman plot and cross-classification analysis.

The estimated daily folate intakes by F-FFQ were 206.2 µg DFE for all 503 studied women and 214.1 µg DFE for the validation subsample. Results of this study are in agreement with previously published data for the population of Serbian women assessed by seven-day food record (228.0 µg/day) [12]. In addition, similar values have been reported in studies conducted in other European countries that assessed folate intake in women using an alternative FFQ (Sweden: 225.0 µg/day [39], The Netherlands: 177.0 µg/day [40], Italy: 222.4 µg/day [41], Norway: 209.0 µg/day [42]), 24 h dietary recalls (Poland: 211.0 µg/day [43], Finland: 205.0 µg/day [44], Greece: 227.2 µg/day [45], Austria: 212.7 µg/day [46]) and 48-h dietary recalls (Spain: 196.9 µg/day [47]). In our study, the main food groups contributing folate intake were identified as vegetables/vegetable products (37.35% of average daily intake), grains/grain products (23.39% of average daily intake) and fruits/fruit products (11.52% of average daily intake). These results are in accordance with the recent comparison of standardized dietary folate intake across ten countries participating in the European Prospective Investigation into Cancer and Nutrition (EPIC) study [48]. A number of national dietary surveys indicate widespread prevalence of suboptimal folate intake among women of childbearing age in Europe highlighting the perspective of achieving folate status associated with lowest risk of folate-related disease (e.g., NTDs), rather than merely preventing evident folate deficiency (i.e., megaloblastic anemia) [14,49,50]. More than 90% of women in our study had folate intake bellow the recommended 400 µg DFE/day. To ensure adequate folate intake and status in general population and particularly in women of reproductive age mandatory folic acid food fortification policies have been established in many countries worldwide, which is not the case in Serbia. Availability of imported and voluntarily fortified foods is rather limited in Serbian market. In addition, results of our study confirm previous findings that folic acid in the form of dietary supplements is rarely consumed by Serbian women of reproductive age [13]. Potential consequences resulting from inadequate folate status on pregnancy outcomes and overall health should be perceived by Serbian public health authorities. It would be wise to consider implementation of educational programs to raise awareness about the significance of this nutrient, as well as nutritional interventions in the form of controlled fortification of selected foodstuffs or targeted supplementation.

It has been suggested that associations between dietary instruments (FFQ and 24 h recalls) estimated by correlation coefficients should be greater than or equal to 0.3, preferably over 0.4 and optimally in the range of 0.5–0.7 [18]. Correlation coefficient observed in the present study between F-FFQ and the average values of three 24 h dietary recalls were *r* = 0.56 (*p* < 0.001) for the total sample, and *r* = 0.57 (*p* <0.001) for the validation group. The strength of these correlations compare favorably to findings reported in other FFQ validation studies. Pauwels et al. [51] developed FFQ to assess usual intake of methyl-group donors and validated it against seven-day food record among Flemish women of reproductive age. They reported a correlation coefficient between two dietary methods of *r* = 0.58. Similar correlation coefficient (*r* = 0.56) was found by Jackson et al. [52] during validation of the 120-item FFQ against repeated 24 h recalls in 70 men and women in Jamaica. Another study compared folate intake assessed by short FFQ and seven-day weighted food record among 37 men and women and reported partial correlation coefficient (controlling for gender) of *r* = 0.53 [53]. In the Northern Sweden, a region without food fortification, FFQ was validated against 24 h recalls where Spearman correlation coefficient between estimates of folate intake based on two methods was *r* = 0.57 for female participants [39]. French et al. [54] developed FFQ to assess the folate intake of women of childbearing age in Canada and validated it against seven-day food record. The observed correlation coefficient between the two methods was *r* = 0.51. Recent study conducted among 67 British women of reproductive age explored the validity of purposefully designed FFQ for the assessment of usual dietary intake of micronutrient methyl donors (folate, choline and betaine) and selected antioxidants. The observed deattenuated correlation coefficient between the FFQ and estimates from three multiple-pass 24 h dietary recalls for folate was *r* = 0.47 for diet only and *r* = 0.80 when supplements were included [55].

In the present validation study serum and RBC folate concentrations were used to validate the dietary estimates of folate intake. Both biomarkers correlated significantly with the intake assessed by dietary methods. Correlation coefficients between F-FFQ and biomarkers were comparable with results of previously published studies for both folate concentration in erythrocytes (r_rbc_ = 0.37 versus 0.34 [24], 0.33 [52] and 0.35 [56]) and in the serum (r_ser_ = 0.28 versus 0.25 [40], 0.20 [52] and 0.26 [57]). Folate serum level is considered to reflect recent intake, while the concentration in red blood cells indicates long-term folate exposure and tissue stores. Taking into consideration that folate accumulates in erythrocytes only during erythropoiesis, RBC folate represents integrative measure of folate intake for the period which corresponds to average lifespan of erythrocytes (i.e., 120 days) [58]. Given that this time-frame is similar to the defined reference period of F-FFQ (i.e., three months), the higher value of correlation coefficient between folate intake assessed by the F-FFQ and the concentration in erythrocytes in relation to the serum is logical. Based on WHO recommendation RBC folate concentration in women of reproductive age should be at least 906 nmol/L so as to ensure optimal prevention of neural tube defects [33]. This value has not been recorded in none of the study participants.

Although correlation analysis is a popular technique which is relatively simple to interpret and compare with results of previously conducted studies, it measures the strength of association between variables, but not the agreement between them [36]. In accordance with recommendations that various statistical approaches should be applied when assessing validity of FFQ, we constructed Bland–Altman plots and performed classification in categories of consumption and status indicators [59]. Bland–Altman plot showed that that the estimates of folate intake obtained by the F-FFQ were comparable to those from repeated 24 h dietary recalls and unlikely to cause systematic bias. Our FFQ performed well in quartile assignment considering that 84% of respondents were correctly classified in the same or adjacent quartile as 24 h recalls and 82% as biochemical status indicators. Similar results of cross-classification analyses were reported in other FFQ validation studies. Percentage of subjects classified within one quartile of folate intake (FFQ versus 24 h recalls) was 84% and 83% for FFQs developed and validated by Johansson et al. [39] and Fayet et al. [56], respectively. In the present study F-FFQ grossly misclassified 4% of women, which is comparable with 4.3% reported by Jackson et al. [52].

Application of the method of triads enabled simultaneous comparison of the F-FFQ with 24 h dietary recalls and biomarkers. In our study, high validity coefficients were determined between the true intake (I) and dietary assessment methods (F-FFQ and repeated 24 h dietary recalls) (ρQI_rbc_ = 0.871 and ρQI_ser_ = 0.814; ρRI_rbc_ = 0.652 and ρRI_ser_ = 0.698) and moderate ones for biomarkers (ρBI_rbc_ = 0.428 and ρBI_ser_ = 0.421). Similar to other studies where the triangulation approach to validation of FFQ has been applied, the obtained validity coefficients for the F-FFQ and 24 h recalls were higher in relation to values for both biomarkers [40,53,60,61,62]. This could be explained by the fact that FFQ and 24 h recalls are methods designed to estimate dietary intake, while the biochemical markers are likely to be influenced by other factors in addition to diet. Our results for validity coefficients are comparable with other studies that used the triads model in validation of FFQ for assessment of folate intake (ρQI_rbc_ = 0.871 versus 0.75, 0.690_man_ and 0.410_women_; ρQI_ser_ = 0.814 versus 0.940, 0.850 _man_, 0.690_women_ and 0.720) [40,53,63], as well as other nutrients such as vitamin D (0.847) [60], α-carotene (0.850) [64], β-carotene (0.760) [37] and vitamin B_12_ (0.950) [65]. This triangulation analyses provided additional valuable insight into F-FFQ’s performance and strengthened the evidence supporting its validity.

The F-FFQ is specific in respect to gender, age and geographic determiners, and, based on our knowledge, this is the first FFQ created and validated for the assessment of folate intake by women of reproductive age in Serbia. Advantages of this validation study include: method of data collection (i.e., interviewer-administered questionnaires according to standardized protocol), application of a detailed food list with incorporated photographs of various food portion sizes and comprehensive statistical validity confirmation with healthy women of reproductive age from different regions of Serbia. Additional advantage is consideration of dietary supplements consumption as well as analysis of folate biomarkers of recent intake and long-term status. Given that there were 503 participants in the total sample, among which 50 provided blood samples for biomarker validation analyses, the sample size in this study was harmonized with recommendations for validation studies [66]. Similar or smaller sample size has been reported for previously conducted studies using the triads method approach (*n* = 50 versus 53 [40], 36 [53], 20 [60] and 28 [64]). Furthermore, narrow 95% confidence intervals for the validity coefficients confirm adequate sample size in our study. It is noteworthy that in this study the so-called Heywood cases (values of validation coefficients greater than 1) have not occurred, which implies absence of random sampling fluctuation between methods or violation of basic model assumptions [20]. Additionally, in order to ensure questionnaire appropriateness regarding cultural and geographic determinants of diet, attention was dedicated to inclusion of locally available food items and traditional dishes that are consumed in Serbia [67]. Due to similar dietary patterns and cultural background, F-FFQ might be useful tool for the assessment of folate intake among other population groups in the Balkans’ region. However, before F-FFQ is used in another population, additional studies are required to explore its validity.

A potential limitation of this study is that the blood sampling for biomarker analyses was conducted only once. Moreover, reproducibility of F-FFQ has not been assessed in this study. However, according to the Altman, method with poor repeatability will never agree well with another method [68]. Good results of F-FFQ and 24 h dietary recalls agreement evaluation suggest that weak reproducibility of this instrument is quite unlikely. Future studies should explore in detail seasonal variations in folate dietary intake by collecting dietary data for every participant in each season over the year, as this was outside the scope of this study due to organizational and financial constraints. However, is unlikely to be a significant limitation since the recent large-scale survey across Europe reported absence of systematic variations for folate intake according to the season of dietary intake collection [48]. Finally, as in most researches in the field of nutrition, general limitations of dietary assessment instruments should be considered. Both dietary methods depend on the memory of respondents and their perception of portion sizes. Nevertheless, potential restrictions were minimized by highly trained interviewers, use of comprehensive Food Atlas and relatively young age of participants.

## 5. Conclusions

Several approaches were used to examine the external validity of the F-FFQ and it performed consistently well. The application of the triads method resulted in high validity coefficients between the true intake and the F-FFQ regardless of biomarker used (ρQI_rbc_ = 0.871 and ρQI_ser_ = 0.814) with narrow 95% confidence intervals and the absence of Haywood cases. Furthermore, Bland–Altman and cross-classification analyses indicated good agreement between methods and satisfactory ranking potential of the F-FFQ. Therefore, considering presented results and similarities with other validation studies, we could conclude that F-FFQ is valid instrument for the assessment of dietary folate intake in women living in Serbia, a country without mandatory folic acid food fortification. In addition, the presented data strongly imply that the Serbian public health strategy should include nutrition initiatives targeting improvement of folate intake and status among women of reproductive age. 

## Figures and Tables

**Table 1:** Daily energy and nutrient intake assessed by the F-FFQ and average of the three 24 h dietary recalls with correlations between the estimates of the dietary intake by applied questionnaires among Serbian women of reproductive age.

Nutrient	Total Sample (*n* = 503)				Pearson Correlation Coefficient	Validation Group (*n* = 50)				Pearson Correlation Coefficient
	F-FFQ		24 h Recalls			F-FFQ		24 h Recalls		
	Mean	SD	Mean	SD	*r*	Mean	SD	Mean	SD	*r*
Energy (kcal)	1724.62	330.02	1734.20	318.50	0.53 **	1727.29	200.36	1718.93	219.40	0.59 **
Carbohydrates (% TEI)	52.23	9.21	50.94	7.23		51.02	5.51	49.88	6.08	
Carbohydrates (g)	225.19	42.72	220.85	59.78	0.51 *	220.31	23.58	214.35	28.21	0.52 **
Fat (% TEI)	27.81	10.61	28.30	7.21		29.34	6.74	31.41	6.30	
Fat (g)	53.29	23.90	54.53	23.72	0.41 **	56.31	14.90	59.99	14.85	0.47 *
Protein (% TEI)	19.52	2.93	20.22	3.50		19.20	2.05	18.49	2.62	
Protein (g)	84.16	13.87	87.66	17.51	0.39 **	82.91	7.38	79.46	8.92	0.40 *
Folate (µg/1000 kcal)	118.98	35.44	121.66	42.73	0.53 **	123.95	32.27	119.90	35.76	0.53 **
Folate (µg)	205.20	61.30	211.00	81.06	0.56 **	214.10	60.78	206.10	65.31	0.57 **

^1^ F-FFQ, Folate Food Frequency Questionnaire; %TEI, percentage of total energy intake; * *p*-value < 0.01, ** *p*-value < 0.001.

**Table 2:** Daily intake levels presented as the median values and 5th and 95th percentiles of twelve food groups and their corresponding contributions to daily folate intake based on repeated 24 h dietary recalls among Serbian women of reproductive age.

Food Groups	Intake of the Food Group (g/Day)			Contribution to Total Folate Intake	
	Median	P5	P95	%	Folate Intake (µg/Day)
Vegetables and vegetable products	175.07	63.95	318.20	37.35	79.66
Grains and grain products	195.91	90.68	338.82	23.39	49.89
Fruits and fruit products	130.75	9.31	378.17	11.52	24.57
Milk and milk products	241.69	15.68	481.12	10.48	22.35
Meat and meat products	95.62	12.31	200.87	5.77	12.31
Nuts, seeds and kernel products	6.00	0.00	66.00	3.01	6.42
Beverages (non-milk)	1.14	0.04	32.59	2.6	5.55
Miscellaneous	5.87	0.00	78.48	2.3	4.91
Eggs and egg products	8.88	0.00	64.10	1.45	3.09
Sea food and related products	0.00	0.00	107.45	1.11	2.37
Sugar and sugar products	17.55	0.75	64.97	0.98	2.09
Fat and oil	11.69	0.00	32.62	0.04	0.09

**Table 3:** Major folate food sources assessed by the F-FFQ and 24 h dietary recalls among Serbian women of reproductive age.

Food	24 h Dietary Recall (*n* = 503)		F-FFQ (*n* = 503)		
	µg DFE/Day (Total Sample)	µg DFE/Day (Consumers Only)	Number of Consumers	% of Consumers	Average Frequency of Consumption
Bread white	15.67	30.11	328	65.21	every day
Beans	10.08	218.38	434	86.28	2–3 times per month
Tomato, raw	9.45	18.75	482	95.83	4–6 times a week
Yoghurt 2.8% mf	7.65	16.05	419	83.30	once a week
Chicken, liver	7.14	251.19	456	90.66	once a week
Peas, green	6.11	93.57	422	83.90	2–3 times per month
Banana, raw	6.06	48.38	492	97.81	2–3 times per week
Egg, hen, whole	5.78	22.45	487	96.82	2–3 times per week
Pepper, red	5.03	51.07	321	63.82	2–3 times per month
Potato	4.98	12.91	225	44.73	2–3 times per month
Strawberries, raw	4.84	26.95	419	83.30	once a week
String beans	4.43	67.11	248	49.30	2–3 times per month
Lettuce, green leaf	4.29	17.98	354	70.38	2–3 times per month
Orange juice, fresh	3.17	62.16	232	46.12	once a week
Cabbage, white	2.71	12.06	393	78.13	2–3 times per month

DFE, dietary folate equivalents.

**Table 4:** Estimated serum and RBC folate concentrations by quartile of intake assessed by F-FFQ among Serbian women of reproductive age.

Quartiles	Folate Intake (µg/Day)	*n*	Serum Folate (nmol/L)			RBC Folate (nmol/L)		
			Mean	95% CI	*p* for Trend	Mean	95% CI	*p* for Trend
1st quartile	<168.43 (147.31)	13	9.54	5.73–13.35		378.29	304.73–451.84	
2nd quartile	168.43–194.12 (182,60)	13	11.48	7.51–15.45		399.05	306.61–491.48	
3rd quartile	194.12–245.62 (217.37)	12	12.18	8.78–15.58		447.27	358.51–536.02	
4th quartile	245.62–362.34 (306.81)	12	15.90	11.51–20.29	0.0139	526.05	444.62–607.48	0.0113

RBC, red blood cells.

**Table 5:** Cross-classification (%) of folate intake into quartiles by the F-FFQ and validation methods (the average of three 24 h recalls and biomarkers of folate status, i.e., serum and red blood cells (RBC folate concentrations) for 50 participants of validation subsample, Serbian women of reproductive age.

Folate Intake/Status Assessed by	Folate Intake Assessed by F-FFQ			
	Same Quartile	Same or Adjacent Quartile	Opposite Quartile	Grossly Misclassified
24 h recall	68	84	12	4
Folate RBC	50	82	14	4
Folate serum	48	82	12	6

## Supplementary Materials

The following are available online at http://www.mdpi.com/2072-6643/9/2/128/s1, Table S1: Food list—F-FFQ.
