# Supplementary material for: Validity of the Food Frequency Questionnaire Assessing the Folate Intake in Women of Reproductive Age Living in a Country without Food Fortification: Application of the Method of Triads
Source: Nutrients. 2017 Feb 13;9(2):128. doi: 10.3390/nu9020128 (PMC5331559; doi:10.3390/nu9020128)
Supplement: Supplementary file 1 [file nutrients-09-00128-s001.docx]

Supplementary Materials: Validity of the Food Frequency Questionnaire Assessing the Folate
Intake in Women of Reproductive Age Living in the Country without Food Fortification: Application of the Method of Triads

Milica Zekovic, Marija Djekic-Ivankovic, Marina Nikolic, Mirjana Gurinovic,
Dusanka Krajnovic and Marija Glibetic

**Table S1.** Food list—F-FFQ.

| **Food Group** | **Foods Included in F-FFQ** |
| --- | --- |
| **Milk, milk products and milk substitutes** | Milk; Milk or soy-based yogurt; Cheese, semi-soft/soft; Cheese, semi-hard/hard; Soy milk; Tofu; Kajmak (traditional dairy spread); Butter |
| **Meat and meat products** | Pork; Beef; Lamb; Veal; Poultry (chicken/turkey); Liver (chicken, beef);  Liver pate; Ham; Sausage; Bacon |
| **Fish and seafood products** | Salmon; Sardines, canned; Tuna, canned; Mackerel; Trout; Catfish; Hake; Seafood mix; Fish pate |
| **Fat and oil** | Sunflower oil; Rapeseed oil; Olive oil; Other oils; Margarine; Lard (pork) |
| **Beverages (non-milk)** | Coffee; Tea; Juice from concentrate with added sugar (orange, apple, apricot, peach etc.); Juice 100% fruit, no sugar added (orange, apple, apricot, peach etc.); Instant vitamin drink (e.g. Cedevita, Multivita etc.) |
| **Eggs and egg products** | Egg, fried/ boiled/steamed/ scrambled |
| **Nuts and seeds** | Walnuts; Hazelnuts; Almonds; Peanuts; Seeds (sunflower, flax, pumpkin) |
| **Vegetables and vegetable products** | Beans; Lentils; Peas, green; String beans; Broccoli; Cauliflower; Kale; Beetroot; Lettuce (iceberg, butterhead, leaf, and romaine); Cabbage;  Pepper; Potato; Spinach; Chard; Carrot; Sweet corn; Sauerkraut; Pickles; Ajvar/pindjur (traditional roasted red pepper spread) |
| **Fruits and fruit products** | Orange; Tangerine; Grapefruit; Lemon; Banana; Apple; Pear; Strawberries; Grapes; Berries (blueberry, blackberry, raspberry); Cherries, sweet/sour; Melon; Watermelon; Plums; Tomato; Dried fruit (prunes/figs, dried/apricots, dried/raisins) |
| **Grains and grain products** | Bread (white/ whole-meal wheat/ rye/multigrain/corn); Rice; Croissant and pastry; Cornmeal; Pasta; Cereals (cornflakes, muesli, oatmeal etc.) |
| **Miscellaneous** | Pie (cheese/dock/spinach); Sweets (candies/cookies/cakes/chocolate); Pizza; Stuffed dock leaves; Prebranac (traditional baked beans); Sun-dried peppers stuffed with beans |

F-FFQ, Folate Food Frequency Questionnaire.
